# Mobile Healthcare and People with Disabilities: Current State and Future Needs

**DOI:** 10.3390/ijerph15030515

**Published:** 2018-03-14

**Authors:** Michael Jones, John Morris, Frank Deruyter

**Affiliations:** 1 Virginia C. Crawford Research Institute, Shepherd Center, Atlanta, GA 30309, USA; john_morris@shepherd.org; 2 Department of Surgery, Duke University Medical Center, Durham, NC 27710, USA; frank.deruyter@duke.edu

**Keywords:** people with disabilities, chronic health conditions, mobile healthcare, mHealth, software applications for smart phones, information and communication technologies

## Abstract

Significant health disparities exist between the general population and people with disabilities, particularly with respect to chronic health conditions. Mobile healthcare—the delivery of healthcare via mobile communication devices—is witnessing tremendous growth and has been touted as an important new approach for management of chronic health conditions. At present, little is known about the current state of mobile healthcare for people with disabilities. Early evidence suggests they are not well represented in the growth of mobile healthcare, and particularly the proliferation of mobile health software applications (mHealth apps) for smartphones. Their omission in mHealth could lead to further health disparities. This article describes our research investigating the current state of mHealth apps targeting people with disabilities. Based on a multi-modal approach (literature review, Internet search, survey of disabled smartphone users), we confirm that people with disabilities are under-represented in the growth of mHealth. We identify several areas of future research and development needed to support the inclusion of people with disabilities in the mHealth revolution.

## 1. Introduction

Over 80% of people with disabilities in the US have one or more chronic conditions that compound the effects of disability on health and function [1]. Moreover, there is growing evidence that people with disabilities, as a group, face significant disparities in accessing healthcare and particularly preventive health and wellness services that may mitigate chronic health conditions [2]. Compared with their non-disabled peers, individuals with disabilities are: (1) less likely to receive recommended preventive health care services (e.g., routine physicals, cancer screenings); (2) at greater risk for poor health outcomes (e.g., obesity, hypertension, fall-related injuries, mood disorders); and (3) more likely to engage in behaviors that put their health at risk (e.g., smoking, inadequate physical activity) [3,4,5].

The health disparities are alarming. For US adults with disabilities, the prevalence of physical inactivity is 120% higher [6], obesity rates are 57% higher [7], smoking rates 47% higher [8], and the prevalence of hypertension is 13% higher [9] than for nondisabled adults. People with disabilities of all ages have more than twice the incidence of diabetes [10,11]. In addition, rates of cardiovascular disease—the leading cause of death in the US—are three times higher among adults with disabilities [12].

*Will growth of mobile healthcare lead to further health disparities for people with disabilities?* Mobile healthcare is being touted as an important new tool for management of chronic health conditions. Mobile healthcare, or mHealth, can be broadly defined as the delivery of healthcare services via mobile communication devices. More specifically, mHealth refers to the delivery, facilitation and communication of health-related information via mobile telecommunication and multimedia technologies—including mobile phones, tablet devices, and wireless infrastructure [13].

Expectations are high for mHealth. About half of patients recently surveyed predict that mHealth will improve the convenience, cost and quality of healthcare in the next three years [14], and 96% of current mHealth app users believe the apps help improve their quality of life [15]. Six in 10 doctors and payers believe that its widespread adoption is inevitable, and 7 in 10 believe health apps will encourage patients to take more responsibility for their health [16]. Supporting these viewpoints, the number of health-related mobile apps is growing at a prodigious rate, from an estimated 40,000 in 2012 to over 165,000 in 2015 [17,18]. Much of this growth has been sparked by the potential of mobile healthcare to support patient engagement and self-management of chronic health conditions. Indeed, patients’ management of their own chronic conditions and active engagement in their healthcare are associated with improved independence and life quality, and reduced healthcare utilization and cost [19,20]. 

Mobile health concepts witnessing high rates of adoption are driven by consumer demand for social health communities, telemedicine, and smartphone apps. The majority (65%) of mHealth smartphone apps are focused on overall health and wellness (e.g., diet and exercise apps), with about 15% of apps focused on self-management of specific conditions (e.g., diabetes). The remaining 20% include apps intended for healthcare providers, (e.g., medication references, assistance with diagnoses), women’s health and pregnancy management, and health information resources (e.g., WebMD) [18].

With a few notable exceptions (autism, mental health, stroke), people with disabilities have not been a primary target for mHealth app development. Concerns have been raised that the proliferation of mHealth could increase health disparities if the apps disproportionately benefit advantaged populations and leave vulnerable populations behind, including people with disabilities. This pattern of widening disparities—termed the “inverse care law”—has been observed following the introduction of other health interventions, particularly those with a social media component [21].

Early evidence suggests that disparities may already exist between disabled and non-disabled populations in adoption of mHealth apps. While smartphone ownership in the US is about equal among disabled and non-disabled adults (71% vs. 68%), the rate of adoption of mHealth apps is lower by half—17% of disabled vs. 34% of non-disabled adults report downloading at least one app that is meant to support their health [22,23].

At present, there is limited information about the representation of people with disabilities in the market for mHealth apps, despite the fact that they experience many of the chronic conditions targeted by these apps. This article describes our preliminary efforts to examine the current state of mHealth for people with disabilities. We examined published literature and online resources to determine the current state of mHealth applications (or “apps”) targeting people with disabilities, and the inclusion of people with disabilities in usability and evaluation studies of mHealth apps, targeting people with disabilities or the general public. We also gathered input from people with disabilities in the US about their experiences with mHealth apps and preferences for future app development. 

## 2. Materials and Methods 

We employed three methods to gather information about the current state of mobile healthcare for people with disabilities. First, we conducted a search for peer-reviewed publications on mobile health and people with disabilities. Our objective was to identify articles describing evaluation studies of mHealth apps developed for people with disabilities. We identified relevant literature from a search of PubMed, Medline, the National Center for Biotechnical Information (NCBI), and the US National Library of Medicine (NLM), using the search terms “mobile health”, “mHealth”, “mobile health apps”, and “disability”. We limited the search to articles published after 2008, the advent of the app-enabled smartphone. We excluded articles that did not include people with disabilities as the target users. While many studies targeted chronic disease and the elderly, they were excluded unless the disability status of study participants was explicitly stated. We also excluded studies addressing validation of assessment tools (e.g., electronic versions of questionnaires), studies identifying or describing needs or barriers to adoption of mHealth apps, articles presenting conceptual or theoretical models for mHealth or opinion/discussion articles in support of or against adoption of mHealth, and articles that described a protocol or study planned or underway but not completed. 

Second, we searched the Internet for “gray literature” and web resources related to mHealth and mHealth apps. We sought information produced by government, business or industry sources and published (on the Internet) outside of traditional academic publishing. We used the Google search engine to search for information pertaining to mHealth and people with disabilities. We used the same search terms as our literature search above (“mobile health”, “mHealth”, “mobile health apps”, and “disability”). We limited the search to English-language content, and excluded peer-reviewed publications, which were discovered in our literature search. In addition to government and industry reports of trends in mHealth, we searched for web resources, such as app curation websites, that provide information for consumers and clinicians to aide in selection of appropriate mHealth apps.

Third, we conducted an online survey of members of our Consumer Advisory Network (CAN) to determine current use and experience with mHealth apps. The CAN is a nationwide sample of over 1500 individuals with disabilities, representing a broad spectrum of vision, hearing, dexterity/mobility, intellectual/cognitive, and communication difficulties. Membership of the CAN reflects the population of non-institutionalized people with disabilities in the US, as compared to the American Community Survey, except that CAN members have higher education and income levels. This results from the fact that our primary source for recruiting CAN members is through the Internet. We believe this is an acceptable variance from the general population of people with disabilities in the US because we are primarily interested in their experiences with information and communication technologies (ICT).

Established in 2001 and maintained through ongoing recruitment efforts, CAN members are frequently called upon to provide insights and feedback on emerging issues of use, usability, needs, and wants related to ICT. Insights from CAN members have formed the empirical basis for reports, presentations, and regulatory filings, and have also contributed directly to proposed research and development projects. CAN members participate in regular surveys including our cornerstone Survey of User Needs (SUN), which has been updated and repeated at regular intervals to provide a longitudinal view of changes in access and use of ICT by people with disabilities [23,24,25,26,27].

We reached out to CAN members via email to encourage them to participate in the online survey on mHealth and disability. The survey was active from February to August 2017. We specifically sought out mobile app users for the survey, and respondents were frequent users of mobile apps with (relatively) high adoption rates of mHealth apps. The survey requested demographic and disability information, and included six force-choice questions (presented in Table 1), with an opportunity to provide additional comments about each question. We also included an open-ended question for respondents to describe their “wish list” of apps for unmet health and wellness needs. 

## 3. Results

### 3.1. Literature Review

Our literature search yielded 91 citations, published between 1 January 2008 and 1 October 2017, which referenced mobile health or mHealth and disability or chronic disease management. Abstracts for all 91 citations were reviewed. A substantial portion (n = 37) of the articles were excluded because their target population was either elderly, had chronic disease or mental health conditions, with no reference to disability in the abstract. Twenty-three articles were excluded as either opinion/discussion articles, or presentations of a theoretical or conceptual framework related to mHealth and not an actual application of mHealth. An additional 23 articles were eliminated based on the following exclusion criteria: validation of a measurement or data collection approach or instrument (6), needs assessment (8) or barrier identification (4) related to mHealth, and descriptions of proposed research or studies in process but not completed (5). 

The remaining eight articles consisted of: four articles providing preliminary assessments of use, acceptability or efficacy of mHealth apps for people with disabilities or their caregivers [28,29,30,31], two articles addressing accessibility issues with use of mHealth apps by consumers with specific impairments (blindness [32] and dexterity impairment [33]), and two review articles addressing the current state of mHealth apps for people with disabilities [34,35]. A brief summary of each article follows.

Parmanto and colleagues [28] describe a mHealth system (iMHere) to support complex self-care tasks for individuals with spina bifida. The system consisted of a smartphone app, a clinical portal, and a communication protocol to support bi-directional data exchange between the app and portal. This article describes the iMHere system and presents findings from a pilot implementation with 14 users with spina bifida over a six-month period. The authors report strong patterns of utilization by 13 of the 14 users but present no findings of clinical efficacy.

Barlott and colleagues [29] describe a study examining the use of short message services (SMS) or text messaging as a resource- and information-sharing tool for caregivers of people with disabilities in a resource-limited Colombian community. Using ethnographic research methods, the study examined qualitative and quantitative evidence of the effects of the messaging system. Caregivers reported a general sense of greater opportunity for social networking, community participation and ability to affect change.

Pavliscsak and colleagues [30] report findings from an investigation of the “mCare” mobile app for community-dwelling, active-duty wounded service members recovering from traumatic brain injury (TBI) and/or post-traumatic stress disorder (PTSD). The app was developed to augment standard care and improve communications. The study followed 95 participants who used the mCare app to communicate with their case manager and platoon sergeant, receive regular notifications (mCare tips, appointment reminders, administrative updates), and complete (seven) weekly questionnaires about their overall functioning. The primary study outcome reported was the level of participant engagement as determined by the number of weekly questionnaires completed. In general, participants responded to over 60% of the questionnaires, which suggests a higher level of engagement than the norm. The authors point to the findings as evidence that mHealth has the potential to improve patient–provider communications.

Jiam and colleagues [31] describe the development of a portable patient profile app (“important information about me” or IIAM) used to store and share healthcare information for people with neurodevelopmental disabilities (NDD). The parents of seven children with NDD participated in a pilot study to test the beta version of the app during a two-month trial. Four of seven participants reported the beta version to be useful, with the greatest limitation in usability being the child’s age and literacy level. All respondents found the app to be visually appealing and easy to navigate. 

The studies above emphasize the dearth of evidence available about the effectiveness of mHealth apps for people with disabilities, as well as the nascent condition of app development for disabled populations. Accessibility of mHealth apps for users with disabilities is another concern. A recent study by Milne and colleagues [32] examined app accessibility for blind users of nine mHealth apps for iPhone. The apps tested were for either glucose or blood pressure monitoring, and all interfaced with an external sensor to measure blood glucose or blood pressure. Existing accessibility guidelines (provided by Apple and Section 508 of the Rehabilitation Act) were used to evaluate the apps, and none of the nine apps were deemed to be accessible to blind users. Yu and colleagues [33] examined the accessibility needs and preferences of users with dexterity impairments in using the iMHere app described previously [29]. Nine participants with varying levels of dexterity impairment participated in a one-week field trial using the iMHere app. As might be expected, a significant negative correlation was observed between degree of impairment and ratio of errors in using the app interface. 

Finally, our literature search uncovered two review articles addressing, at least obliquely, the current state of mHealth apps for people with disabilities. Vandelanotte et al. [34] provide an overview of the state of evidence for the use of eHealth and mHealth to improve physical activity and nutrition behaviors in general and special populations. The authors distinguish between eHealth (use of information and communication technology, especially the Internet, to improve health) and mHealth (medical and public health practice supported by mobile devices). In reviewing eHealth/mHealth research with special populations, the authors report finding only one article involving people with disabilities (a study of an Internet-based intervention to increase physical activity in adolescents with cerebral palsy [36]), and conclude that recruitment of people with disabilities into eHealth/mHealth trials must be particularly challenging.

Kao and colleagues [35] published a recent review, intended for physicians in rehabilitation medicine. The review of consumer mHealth apps provides a good overview of the current state of the field and (regulatory, evidence, and privacy/security) barriers to future adoption. However, the review is noticeably light in covering mHealth apps relevant to the field of rehabilitation medicine and disability. Only one app (Pt Pal Pro) is described, with no evidence presented about efficacy. The app allows rehabilitation physicians and other therapists to prescribe home exercise programs for their patients and track patient adherence and progress.

### 3.2. Internet Search

Our internet search uncovered several recent publications with useful insights into the current landscape of mHealth apps for people with disabilities. The IMS Institute for Healthcare Informatics published a report in 2015 describing use, evidence and remaining barriers to mainstream adoption of mHealth apps by patients and healthcare providers [18]. The report compares findings from a 2013 and 2015 study of mHealth apps available for download from the US Apple iTunes store and Google (Android) Play marketplace. In the 2015 study, over 165,000 mHealth apps were identified. Through review and selection criteria, including frequency of downloads, 26,864 apps were selected for further study as representative of the most widely used mHealth apps by consumers. 

Mobile health apps are generally divided into two broad categories: those that facilitate overall health and wellness, and those focused on disease management. Health and wellness apps accounted for about two-thirds of those examined in the IMS study, and address fitness (36%), lifestyle and stress management (17%), and diet and nutrition (12%). Only 15% of apps targeted a specific disease or condition, most commonly diabetes, blood pressure and mental health conditions. Mental health apps (as categorized by IMS) comprised almost one-third of the disease specific apps; the most commonly addressed conditions were autism, anxiety, depression, ADHD (Attention Deficit Hyperactivity Disorder), and Alzheimers’ disease. In total, disability-focused apps (including mental health) accounted for only about 2% of all mHealth apps examined.

Specialized curation websites offer another source of information about the current state of mHealth apps, and the limited number of apps targeting people with disabilities. Four of the most popular sites based on usage areAppscript (https://www.appscript.net/), Happtique (http://www.happtique.com/), MyHealthApps in the UK (http://myhealthapps.net/) and Wellocracy ( http://www.wellocracy.com/), a venture of PARTNERS Healthcare Center for Connected Health in Boston. The Wellocracy site is focused on health and wellness, and does not include disease management apps. The site provides information about both mHealth apps and wearable or other tracking/recording devices. Apps are categorized into health and fitness, diet and nutrition, and sleep and mood management. None of the apps or devices featured target people with disabilities specifically.

MyHealthApps offers the most comprehensive list of apps available online, and includes apps originating in the US and throughout Europe. There is no formal evaluation or scoring of apps, but all are recommended by healthcare communities, including consumers, patients, caregivers, patient groups, and charities and other not-for-profit organizations. The site lists 21 categories of apps and most are focused on management of specific health conditions, rather than overall health and wellness. A search for the term “disability” yields 63 apps. However, almost all of these apps are not related to health or health care. Instead, they include accessibility apps (e.g., text-to-speech or speech-to-text apps, currency readers), communication apps (e.g., augmentative communication software, sign language translators), and informational apps to support community living (e.g., locating an accessible restroom or lodging). A handful of apps are intended to assist with management of a specific disability (e.g., Parkinson’s, cerebral palsy) and include only general information related to health or healthcare (e.g., how to prepare for a visit with you doctor).

Both AppScript and Happtique curate apps based on proprietary evaluation protocols, including input from healthcare professionals and patients. The AppScript website provides information on over 700 mHealth apps, categorized as: Diet & Nutrition, Disease Specific, Exercise, Lifestyle & Stress, Medication Reminders & Info, and Women’s Health & Pregnancy. There are 274 disease specific apps and the following disabilities are included: Alzheimers (8 apps), autism and communication disorders (5), cognitive and memory problems (6), MS (4), rheumatoid arthritis (2), and Parkinson’s disease (1). The Happtique “App Boutique” was created by SocialWellth and lists over 800 apps curated based on a detailed evaluation and certification protocol. Five broad categories are included on the website: Conditions (e.g., allergies, autism, chronic pain, cognitive impairment, substance abuse), Fitness, Food (diet and nutrition), Lifestyle (e.g., relationships, spirituality, recreation and leisure), and Wellness (e.g., alternative medicine, medication adherence, stress management). There are a handful of targeted disability apps (autism, multiple sclerosis (MS), Parkinson’s, PTSD).

Thus, while curation sites provide some assurances about the quality and usability of recommended apps, those targeting people with disabilities represent a very small percentage of the curated offerings. Moreover, the evaluation criteria used by sites to score or certify apps do not address accessibility and utility of the app from the perspective of people with disabilities.

There are also numerous web resources supported by industry, government agencies, and advocacy organizations that provide information about mobile apps for people with disabilities. Table 2 provides information we compiled from 11 websites that include directories of mobile apps for use by people with disabilities. Nine websites are from the US and one each from Australia and Ireland. Two of the websites (the Federal Communication Commission’s (FCC) Accessibility Clearing House and AppAdvice) have directories for different disability groups, and are listed separately. These sites provide information on over 3200 apps (although some of the “apps” are actually websites or other internet-based resources). Most of the apps listed are designed for people with disabilities, but few are mHealth apps. Most of the apps related to health and wellness topics are for more general use (e.g., diet and nutrition, exercise trackers, medication reminders), and may not be usable by people with disabilities. In fact, we found only 56 disability-specific mHealth apps focused on health and wellness.

### 3.3. Survey of User Needs and Preferences for Mobile Health

Our preliminary research offers additional insights concerning mHealth apps and people with disabilities. A total of 377 respondents completed the mHealth survey. Average age of respondents was 54 (SD = 14.5 years); 53% were female and 74% were white/Caucasian. About half (47%) of respondents reported household income below $50,000. Respondents were asked to identify whether they had difficulties in any of nine general functional categories (Table 3). Most reported on average as having two functional limitations/difficulties with the most common being difficulty walking, climbing stairs and difficulty hearing. Respondents reported using a wide variety of mHealth apps. Exercise and activity tracking apps were the most commonly reported mHealth app, used by 40% of respondents. Diet and nutrition apps were used by 27% of respondents and lifestyle management (stress management, sleep quality) apps by 17% of respondents.

Two key questions in the mHealth survey focus on: (1) ease of finding usable and effective mHealth apps, and (2) satisfaction with the use of mHealth apps. Respondents were asked to rate ease or difficulty on a 5-point scale from very difficult to very easy. Ratings of “ease of finding a usable and effective mHealth app” were summarized into a single “Ease index” by assigning values of 1 to 5, respectively, to the responses “very difficult” to “very easy”. These values were multiplied by the number of respondents who reported each level of ease/difficulty. The product of this operation was then divided by the highest possible value that would result if all respondents rated their ease/dificulty in funding a usable app as “very easy”.

Respondents were asked to rate their satisfaction with usability of mHealth apps on a 5-point scale, from very dissatisfied to very satisfied. A Satisfaction index was calculated using the same methodology as with the Ease index. Table 4 presents the Satisfaction and Ease index for each disability type and overall. Feedback was mixed concerning the ease or difficulty in locating a suitable mHealth app, and overall satisfaction with existing mHealth apps. Only 46% of respondents reported that it was easy/very easy to locate a suitable app and the same proportion indicated they were satisfied or very satisfied with use of the app. There were modest differences by disability group—individuals with impairments related to using their arms and fatigue/stamina reported lower satisfaction, and those with impairments related to seeing, using arms, walking, and fatigue/stamina reported greater difficulty in locating mHealth apps.

This difficulty/dissatisfaction was also reflected in the comments about accessibility and usability issues participants experienced with the use of mHealth apps. Of the comments received concerning accessibility/usability issues with apps: 26% were related to the difficulty in setting up and using apps consistently; 17% commented on problems with the accuracy of apps that involved monitoring or measurement; and 10% of comments related to the lack of apps that adequately account for disability. For example, respondents with activity limitations requested that diet and exercise apps more accurately measure activity levels (e.g., when using a wheelchair or other mobility aid) or allow for adjustments to diet/nutrition goals to suit their more limited caloric intake needs. Many respondents requested compatibility with assistive technology (e.g., screen reader) or alternatives to manual keypad entries (e.g., difficulty using zoom or keypad gestures; using radio buttons; an “undo” function). Blind respondents indicated the need for captioning in apps using video.

When asked about “wish list” items for mHealth app development, a common theme was the need for an app to help manage health information, symptom tracking, and communications with healthcare providers about a person’s disability and its effects on health. Many respondents wished for apps that were better integrated—an “all-in-one” app that could be used to track exercise, diet, medications, and biologic data (e.g., heart rate, blood glucose level, blood pressure) without the need to interface with other devices. There were also numerous requests for disability-specific apps for exercise, fitness and diet tracking.

Finally, respondents overwhelmingly (89%) supported the idea of a curation website with information about mHealth apps suited for people with disabilities. They particularly supported the value of app reviews by people with similar disabilities as a method to locate a suitable app.

## 4. Discussion

Taken together, results from our three-pronged investigation provide several insights about the current landscape of mHealth and people with disabilities. First, our literature review supports the fact that mHealth development and application for people with disabilities is in its early stages; only a handful of articles were identified with relevance to mHealth and disability, and these described a small number of mHealth apps with virtually no evidence of effectiveness on health outcomes of people with disabilities. We did limit our search to published articles that referenced people with disabilities as target users for the apps being addressed, and indeed many “mainstream” mHealth apps may be usable and effective for people with disabilities. However, we have no way of determining their relevance unless people with disabilities are included in studies of their usability or effectiveness. Second, our Internet search produced useful evidence supporting the growth of mHealth in general but also reinforced the fact that apps targeting people with disabilities make up only a fraction of mHealth apps on the market. 

Third, our survey of disabled users of mobile apps indicated a high adoption rate (40%) of mHealth apps among these users, but also pointed to difficulties in locating suitable apps for disabled users, problems with accessibility of apps, and concerns about the accuracy or relevance of content in mainstream mHealth apps for disabled users. One caveat to the interpretation of these findings is the nature of our survey respondents. Although representative in terms of disabling conditions, we specifically targeted respondents who are smartphone and mobile app users. As a result, the education and income level of survey respondents is not representative of the US population of people with disabilities. Furthermore, since this was a convenience sample, it is skewed toward a greater proportion of white/Caucasian respondents (84%) and fewer African American (5%) and Hispanic/Latino (4%) respondents than the US population (72.4% white/Caucasian; 12.6% African American; 12.8% Hispanic/Latino).

These insights suggest the need for additional research to: (1) determine the availability, usability, and clinical effectiveness of mHealth apps for people with disabilities, and (2) identify priority needs for mHealth app development, based on the gap between healthcare needs and available/accessible apps to address those needs.

Future mHealth app development must address the priority needs and unique challenges of people with disabilities in accessing and using mHealth apps. Our review of the current landscape and input from consumers suggests that three types of mHealth apps are needed. First, mainstream health and wellness apps and those for managing chronic health conditions and risk factors (e.g., diabetes, cardiovascular disease, obesity) may need to be “recalibrated” for use by people with disabilities. For example, diet and exercise apps for people with paralysis need to be tailored to their nutritional needs, physical capabilities, and accessibility requirements for use of exercise equipment (e.g., from a wheelchair). The content and method of content delivery of a diabetes management app may need to be adapted to meet the cognitive capacity of an individual with acquired brain injury.

Second, mHealth apps are needed that target health conditions or risks unique to people with disabilities. While apps exist addressing specific disability conditions such as Parkinson’s and MS, most of these are informational in nature or provide limited functionality, such as symptom tracking. There are numerous health conditions or risks unique to disabling conditions that would benefit from mHealth apps. For example, apps targeting pressure ulcer prevention behaviors or management of respiratory functioning for people with paralysis could be important to prevention of secondary conditions.

Third, accessibility interfaces or add-ons that work with mHealth apps may be needed for some disabled users. We noted previously the finding that none of the tested apps for glucose and blood pressure monitoring were usable by people who are blind [32]. Assistive technology solutions (e.g., screen reader) may be needed so that clinically effective mHealth apps are accessible and usable by people with disabilities. mHealth apps need to be designed to accommodate the accessibility needs of disabled users, including those with limitations in vision, hearing, dexterity and motor control, speech, and cognitive abilities.

In addition to development efforts in these areas, work is needed to help people with disabilities in locating apps that are suitable to their needs. As noted in our survey findings, there is strong support for a curation website with information about mHealth apps suited for people with disabilities. Accessibility ratings of apps and reviews by people with similar disabilities were viewed as especially helpful as methods to locate a suitable app.

Finally, our findings support the need for additional research to evaluate the effectiveness of mHealth apps in improving the health outcomes of people with disabilities. This concern is by no means limited to disabled users of mHealth. Given the recent emergence of mHealth, empirical evidence of the efficacy of apps remains limited. Traditional approaches to generating this evidence (with the randomized trial as the gold standard) are poorly suited for a constantly-evolving technology endeavor with considerable heterogeneity in its application. We echo Kao and colleagues [36] in challenging the field to develop evidence-generating mechanisms that can match the rigor of randomized trials while keeping pace with the speed of evolution of mHealth innovations.

## 5. Conclusions

Our findings demonstrate the considerable opportunity, need, and largely inadequate response to date, of emerging mHealth solutions for people with disabilities. Substantial health disparities support the need for effective healthcare and health maintenance interventions for people with disabilities. Responding to this need requires a sustained, focused, and well-resourced effort, including: (1) research: to identify priority needs of people with disabilities for mHealth app development, especially those who already face health disparities; (2) development: aimed at designing, deploying, and validating new mHealth solutions that respond to the most pressing needs of people with disabilities; and (3) a knowledge translation: effort to assist consumers and healthcare providers in identifying accessible and effective mHealth apps that address health disparities and improve health outcomes for people with disabilities. 

## Figures and Tables

**Table 1:** mHealth Survey questions.

1.	Which of the following types of health and wellness apps do you use?
	Fitness, exercise, and physical activity
	Diet, nutrition, health eating
	Lifestyle and stress management, including sleep quality
	Other areas related to general health and wellness
2.	Do you use any apps that help you manage one of the following health conditions or areas?
	None—I do not use apps for any of the following
	Medication management or reminders
	Obesity/weight control
	Diabetes Heart and circulatory conditions, including blood pressure Mental or psychological health, including anxiety and depression Allergies
	Managing thinking and remembering difficulties Chronic pain Other chronic conditions or disability Women’s health and pregnancy
3.	In general, how satisfied are you with the use of these apps to manage your health, wellness or specific condition?
	Very satisfied
	Satisfied
	Neutral
	Dissatisfied
	Very dissatisfied
4.	How easy or difficult was it for you to find mHealth apps that work well for you?
	I have never searched for an mHealth app
	Very easy
	Easy
	Neutral
	Difficult
	Very difficult
5.	If it existed, would you use a website that provides information and recommendations for mHealth apps specifically for people with disabilities? Yes or No
6.	Would it be helpful to have a website that provides reviews or feedback about apps from users with conditions similar to your own? Yes or No

**Table 2:** mHealth apps available on resources websites for people with disabilities.

Website Name	Disability Category	# of Apps on Site	# of mHealth Apps on Site	Names of Apps
FCC’s Accessibility Clearing House (www.ach.fcc.gov)	Blind/Visually Impaired	88	0	
FCC’s Accessibility Clearing House	Cognitive	56	6	Behavior Status, In Case of Emergency (ICE) personal health information; PE (Prolonged Exposure) Coach; PTSD Coach; Seizure Log; Small Talk Pain Scale
FCC’s Accessibility Clearing House	Mobility	40	3	ICE; RxMindMePerscription; Seizure Log
GARI App Database (https://www.gari.info/findapps-results.cfm)	All	117	3	Tetra Alarm; Hear and Tinitus; MIMI MearingTest
Apps for the Deaf and Hearing Impaired (www.appadvice.com)	Deaf/HoH	14	0	
iOS Apps Developed Specifically for Blind or Low Vision Users (www.applevis.com)	Blind/Low Vision	112	1	CrowdViz
Orion ISO (www.orioniso.com)	All	21	1	My Emergency Info
Friendship Circle (www.friendshipcircle.org)	Autism/Learning Disabilities	7	1	Behavior Tracker Pro
AFB’s Collection of Accessible Apps for Android (www.afb.org/afbpress/pub.asp?DocID=aw140303)	Blind	10	0	
Assist Ireland (www.assistireland.ie)	All	103	5	Pill Reminder; Med Coach; iFall; Fall Alert; Fall Detection
Choice (www.choice.com.au)	All	8	0	
Living Well with a Disability (www.livingwellwithadisability.org)	All	5	0	
Disabled-World (www.disabled-world.com)	All	106	1	WebMD Webscape (for healthcare providers)
Bridging Apps (www. search.bridgingapps.org/apps)	All	3245	35	ICE; Emergency: Alerts notifications, and preparation; Depression and mood management; Anxiety management (relaxation, mindfulness, deep breathing); CareZone (disease management app for caregivers); Bipolar Disorder, ADHD, PTSD, OCD management; Track-It seizure log; Exercise Buddy for Autism and children with DD; Bowel Mover Pro for IBS; Birdhouse for Autism (behavior, health and daily living manager for parents); Moving Forward problem-solving app for chronic disease management; PumpPartner for managing baclofen pump; SlowControl vibrating fork app to slow rate of eating; Grey Matters for caregivers managing dementia; Feeding Tube Kids for managing tube feedings; Connections information and referral app for caregivers of special needs children
		3932	56	

**Table 3:** “Which of the following types of health and wellness apps do you use? (Check all that apply)”, by disability type.

	Fitness	Diet	Lifestyle	Other
Difficulty concentrating, remembering, deciding	60%	24%	22%	22%
Frequent worrying, nervousness, or anxiety	50%	24%	24%	29%
Difficulty seeing	44%	23%	23%	29%
Difficulty hearing	45%	30%	17%	18%
Difficulty speaking so people can understand you	47%	41%	29%	29%
Difficulty using your arms	30%	30%	22%	30%
Difficulty using your hands and fingers	44%	31%	26%	21%
Difficulty walking or climbing stairs	37%	28%	17%	22%
Difficulty with fatigue/limited stamina	40%	30%	23%	26%
All respondents	40%	27%	17%	20%

**Table 4:** Satisfaction with mHealth apps and ease of finding mHealth apps that work for me.

Disability Type	Satisfaction Index	Ease Index
Difficulty concentrating, remembering, making decisions	3.51	3.38
Frequent worrying, nervousness, anxiety	3.68	3.70
Difficulty seeing	3.31	2.98
Difficulty hearing	3.57	3.23
Difficulty speaking so people can understand you	4.00	3.43
Difficulty using your arms	3.19	2.99
Difficulty using your hands and fingers	3.24	3.05
Difficulty walking or climbing stairs	3.29	2.97
Difficulty with fatigue/limited stamina	3.12	2.95
All respondents	3.46	3.25
